# Antagonizing S1P_3_ Receptor with Cell-Penetrating Pepducins in Skeletal Muscle Fibrosis

**DOI:** 10.3390/ijms22168861

**Published:** 2021-08-17

**Authors:** Angela Corvino, Ida Cerqua, Alessandra Lo Bianco, Giuseppe Caliendo, Ferdinando Fiorino, Francesco Frecentese, Elisa Magli, Elena Morelli, Elisa Perissutti, Vincenzo Santagada, Giuseppe Cirino, Elisabetta Granato, Fiorentina Roviezzo, Elisa Puliti, Caterina Bernacchioni, Antonio Lavecchia, Chiara Donati, Beatrice Severino

**Affiliations:** 1 Department of Pharmacy, School of Medicine, University of Naples «Federico II», Via D. Montesano 49, 80131 Napoli, Italy; angela.corvino@unina.it (A.C.); ida.cerqua@unina.it (I.C.); alessandra.lobianco@unina.it (A.L.B.); caliendo@unina.it (G.C.); fefiorin@unina.it (F.F.); frecente@unina.it (F.F.); elisa.magli@unina.it (E.M.); elena.morelli@unina.it (E.M.); perissut@unina.it (E.P.); santagad@unina.it (V.S.); cirino@unina.it (G.C.); elisabetta.granato@unina.it (E.G.); roviezzo@unina.it (F.R.); antonio.lavecchia@unina.it (A.L.); 2 Department of Experimental and Clinical Biomedical Sciences “Mario Serio”, University of Florence, Viale GB Morgagni 50, 50134 Firenze, Italy; elisa.puliti@unifi.it (E.P.); caterina.bernacchioni@unifi.it (C.B.); chiara.donati@unifi.it (C.D.)

**Keywords:** sphingosine-1-phosphate, S1P_3_ receptor antagonist, skeletal muscle fibrosis, molecular dynamics simulations

## Abstract

S1P is the final product of sphingolipid metabolism, which interacts with five widely expressed GPCRs (S1P_1-5_). Increasing numbers of studies have indicated the importance of S1P_3_ in various pathophysiological processes. Recently, we have identified a pepducin (compound KRX-725-II) acting as an S1P_3_ receptor antagonist. Here, aiming to optimize the activity and selectivity profile of the described compound, we have synthesized a series of derivatives in which Tyr, in position 4, has been substituted with several natural aromatic and unnatural aromatic and non-aromatic amino acids. All the compounds were evaluated for their ability to inhibit vascular relaxation induced by KRX-725 (as S1P_3_ selective pepducin agonist) and KRX-722 (an S1P_1_-selective pepducin agonist). Those selective towards S1P_3_ (compounds **V** and **VII**) were also evaluated for their ability to inhibit skeletal muscle fibrosis. Finally, molecular dynamics simulations were performed to derive information on the preferred conformations of selective and unselective antagonists.

## 1. Introduction

Bioactive lipids are important mediators of cellular signaling in eukaryotic cells. S1P is the final product derived from the metabolism of plasma membrane glycosphingolipids and sphingomyelin [1]. Red blood cells, endothelial cells and platelets are the major sources of S1P in plasma. S1P acts through five known subtypes of heptameric G-protein-coupled receptors (GPCR), namely S1P_1_–S1P_5_ (S1PR). The activation of these receptors can promote opposite cellular responses [2]. This apparent discrepancy can be readily explained by the fact that each subtype is coupled to a different G-protein. Indeed, S1P_1_ is coupled to Gαi, while S1P_2_ and S1P_3_ to Gαi, Gα12/13 and Gαq. By contrast, S1P_4_ and S1P_5_ can only activate Gαi and Gα12/13 [3]. Recent evidence indicates that the S1P signaling axis contributes to the development and maintenance of the fibrotic process [4].

Fibrosis is a pathological condition that can affect every organ since it is the consequence of a persisting inflammatory and tissue remodeling condition. In this inflammatory environment, the deposition of extracellular matrix (ECM) prevails, and organ function is gradually lost. Transforming growth factor β (TGFβ), synthesized by multiple cell types recruited at the site of inflammation, represents a crucial mediator. In different fibrotic models, extensive crosstalk has been shown between the TGFβ and S1P signaling axes. S1P_3_ has been involved in fibrosis development in different tissues, such as skeletal muscle, liver, and kidney [5,6,7]. In this context, the S1P_3_ receptor and its downstream signaling have been identified as candidate targets for myoblast transdifferentiation into myofibroblasts [5,8]. Thus, selective antagonists of the S1P_3_ receptor could be useful to better define the role of S1P_3_ in fibrosis, as well as to develop a novel therapeutic strategy to treat inflammation-associated fibrosis.

Pepducins specifically target the intracellular loops, acting as allosteric modulators of GPCR activity [9]. Moreover, an increasing amount of data indicate how these cell-penetrating lipopeptides can access receptor conformations not available to orthosteric ligands. Using this approach, we have synthesized a pepducin-based S1P_3_ antagonist, namely KRX-725-II (Myristoyl-GRPYDAN-NH2) [10,11]. Here, to overcome the poor selectivity between S1P_1_ and S1P_3_, due to the high structural homology between the two receptors [12], we have synthesized several derivatives of KRX-725-II. The synthetic strategy is focused on the aromatic residue of the sequence, Tyr4, aiming to introduce molecular constraints to achieve better selectivity. The new molecular entities (Table 1) have been evaluated for their selectivity profile by using mouse aortas. This allowed us to identify compounds **V** and **VII** (embodying respectively L- and D-Tic) as the most selective S1P_3_ antagonists. These two compounds have been tested on murine skeletal myoblasts by using TGFβ1 as stimulus and were studied by molecular dynamics (MD) simulations to explain the higher selectivity observed.

## 2. Results

### 2.1. Design

We have already identified a pepducin (compound KRX-725-II in Table 1) acting as an S1P_3_ receptor antagonist [10]. Here, we have synthesized a series of new derivatives to optimize the activity and selectivity profile toward S1P_3_. 

**Figure 1:**
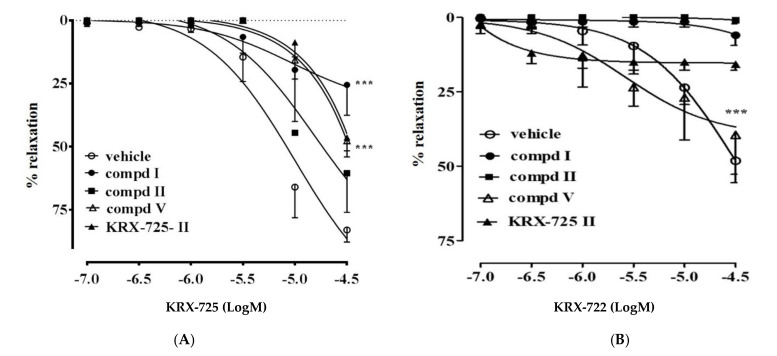
Panel (**A**): Pretreatment of aortic rings with compounds **I**, **II**, **V** and KRX-725-II abrogates KRX-725-induced vasorelaxation. Panel (**B**): Compound **V** does not affect KRX-722-induced vasorelaxation. Data are expressed as mean ± s.e.m *** *p* < 0.001 vs. vehicle as assessed by two-way ANOVA followed by Bonferroni’s multiple comparison tests.

To this end, the efficacy of the new compounds was compared to the S1P_3_ (e.g., KRX-725) and S1P1 (e.g., KRX-722) agonists [11]. As expected, KRX-725-II significant inhibited KRX-725-induced relaxation (Figure 1A), as well as KRX-722-induced vasorelaxation (Figure 1B), demonstrating a limited selectivity.

The Ala-scan, previously conducted on KRX-725, suggested its high tolerance to amino acid substitutions [10], and directed the research towards more selective S1P3 antagonists by chemical modification of the native sequence. Firstly, we focused our attention on Tyr, in position 4, and synthesized a new series of compounds to verify if: (1) the presence of Tyr is determinant in the interaction with S1P_3_; (2) the introduction of molecular constraints could address the selectivity towards one specific receptor. We inserted in position 4 natural (e.g., Phe, Trp and Pro) and unnatural aminoacids (e.g., D-Tyr, p-F-Phe, 4-CH_3_SO_2_NH-Phe, L-Tic, D-Tic and L-Pip). Among them, L-Tic and D-Tic were chosen as L- and D-Phe mimetics. 1,2,3,4-Tetrahydroisoquinoline-3-carboxylic acid (Tic) is a chimaera of Phe and pipecolic acid (Pip), where χ1 can adopt the two-gauche conformation, but not the trans conformation [13]. The sequences of the newly synthesized compounds are reported in Table 1.

### 2.2. Vascular Activity

S1P receptor activation was assessed ex vivo by using aortic rings to define S1P_3_ selectivity. Exogenous administration of S1P induces a significant and endothelium-dependent vasorelaxation in mouse aortic rings precontracted with phenylephrine (PE). This effect is mediated by both S1P_1_ and S1P_3_ receptors. To assess selectivity, pepducin-based and orthosteric agonists for S1P_1_ (KRX-722 and SEW2871) and S1P_3_ (KRX-725 and CYM5541) were used. The biological evaluation was first conducted on compounds **I–VI**. Among these, compounds **I**, **II** and **V** demonstrated similar or increased activity when compared to KRX-725-II in inhibiting KRX-725-induced vasorelaxation (Figure 1A). Only the compound **V** did not affect KRX-722-induced relaxation (Figure 1B). Indeed, both compounds **I** and **II**, similarly to KRX-725-II, significantly inhibited S1P_1_-mediated relaxation, demonstrating a limited selectivity versus S1P_3_.

**Figure 2:**
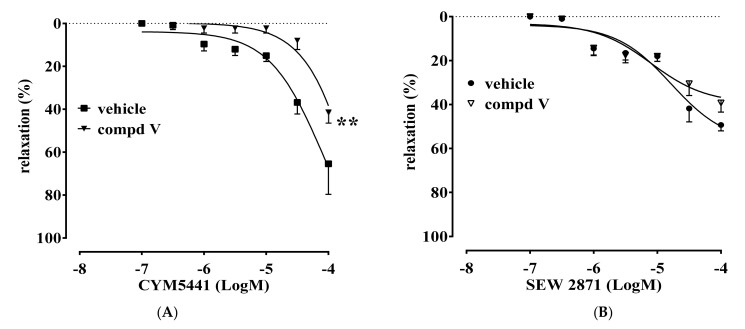
Panel (**A**): Compound **V** significantly inhibited CYM5541-induced vasorelaxation. Panel (**B**): Pretreatment of aortic rings with compound **V** did not affect SEW2871-induced vasorelaxation. Data are expressed as mean ± s.e.m ** *p* < 0.01 vs. vehicle as assessed by two-way ANOVA followed by Bonferroni’s multiple comparison test.

To further confirm our data, we evaluated the ability of compound **V** to interfere with the vasodilator response elicited in vitro by commercially available orthosteric-selective agonists for either S1P_1_ (SEW2871) or S1P_3_ (CYM5541). In accordance with the previous data, we found that compound **V** significantly and selectively inhibited only CYM5541-induced vasorelaxation (S1P_3_; Figure 2A), while it did not affect SEW2871-induced effects (S1P_1_; Figure 2B).

The obtained results prompted us to replace L-Tic with D-Tic to obtain compound **VII,** in order to verify the presence of a possible stereospecific interaction with the target. Moreover, to isolate the backbone effects imposed by L-Tic (compound **V**), we prepared the L-Pip (compound **VIII**) and L-Pro (compound **IX**) analogues. 

**Figure 3:**
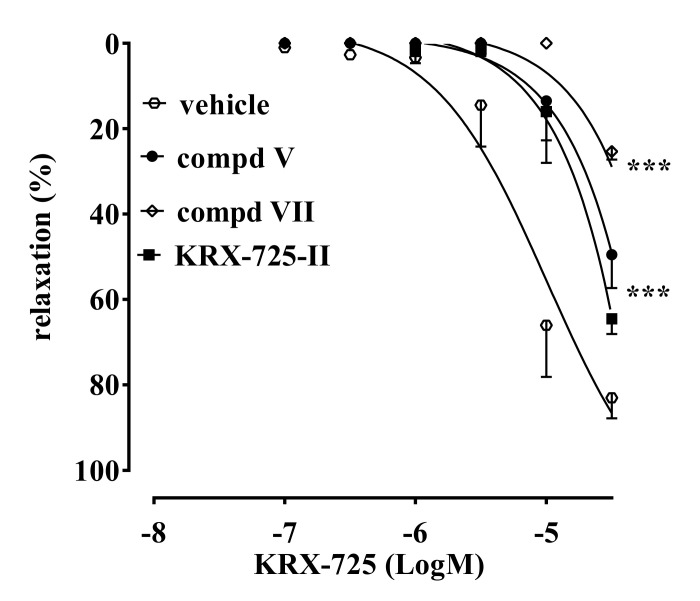
Compound **VII** significantly inhibited KRX-725-induced vasorelaxation when compared to KRX-725-II or compound **V**. Data are expressed as mean ± s.e.m *** *p* < 0.001 vs. vehicle as assessed by two-way ANOVA followed by Bonferroni’s multiple comparison test.

Compounds **VIII** and **IX** were devoid of antagonistic activity. On the other hand, compound **VII** (Figure 3) showed an antagonist profile similar to compound **V** and KRX-725-II. However, compound **V** inhibited about 70% of the KRX-725-induced relaxation, versus the 50% inhibition induced by the other two compounds tested.

### 2.3. Anti-Fibrotic Activity

It has been previously shown that the S1P_3_ blockade significantly decreases the TGFβ1-induced transdifferentiation of myoblasts into myofibroblasts, diminishing the expression level of the fibrotic markers alpha-smooth muscle actin (αSMA) and smooth muscle alpha 22 (SM22α) [7]. The ability of the selected compounds (**V** and **VII**) to modulate S1P_3_ receptor activation was assessed by determining their ability to significantly reduce the profibrotic action of TGFβ1 in C2C12 myoblasts.

**Figure 4:**
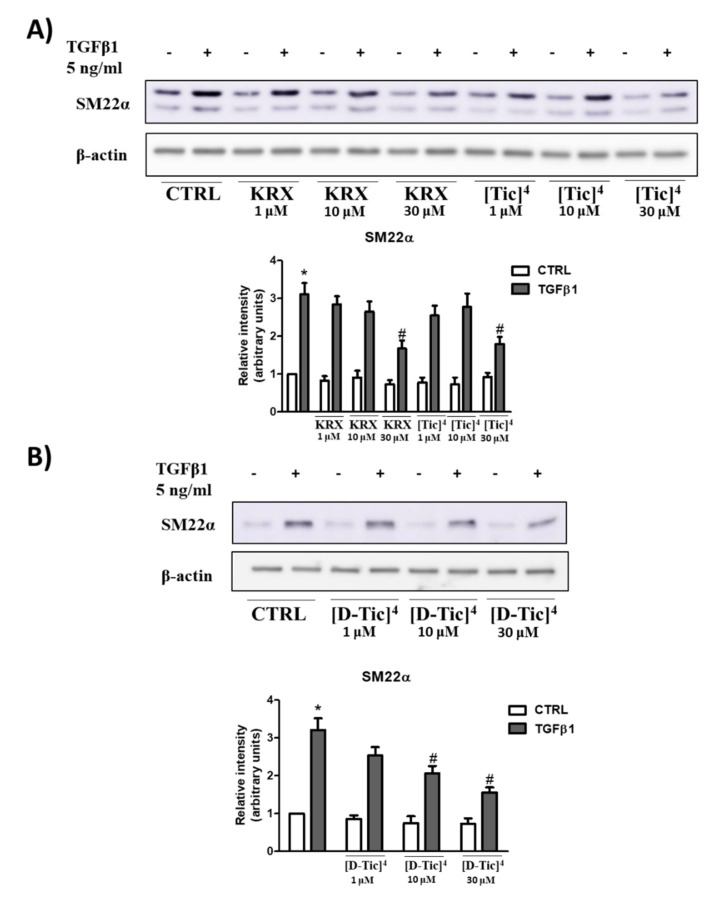
Subconfluent C2C12 myoblasts were pre-incubated with S1P_3_ antagonists KRX-725-II (KRX), [L-Tic]^4^-KRX-725-II ([Tic]^4^) (**A**) or [D-Tic]^4^-KRX-725-II ([D-Tic]^4^) (**B**) at the indicated concentration for 1 h before being stimulated with 5 ng/mL TGFβ1 for 48 h. The content of SM22α was analyzed by Western blotting in cell lysates. Representative blots are shown. The histograms represent the densitometric analyses of three independent experiments. Data are the mean ± SEM and are reported as protein expression normalized to β-actin, in terms of fold change over control. TGFβ1 significantly increases the expression levels of fibrotic marker SM22α (*, *p* < 0.05). The blockade of S1P_3_ by 30 μM KRX-725-II, 30 μM [Tic]^4^-KRX-725-II and 10 μM and 30 μM [D-Tic]^4^-KRX-725-II significantly inhibits TGFβ1’s effect (#, *p* < 0.05).

Subconfluent C2C12 myoblasts were pre-treated for 1 h with different concentrations of KRX-725-II, [Tic]^4^-KRX-725-II (compound **V**) and [D-Tic]^4^-KRX-725-II (compound **VII**) (1 μM,10 μM and 30 μM) before being challenged with 5 ng/mL TGFβ1 for 48 h. As shown in Figure 4, both compounds inhibited the expression of the fibrotic marker SM22α induced by TGFβ1, as determined by Western blot analysis.

**Figure 5:**
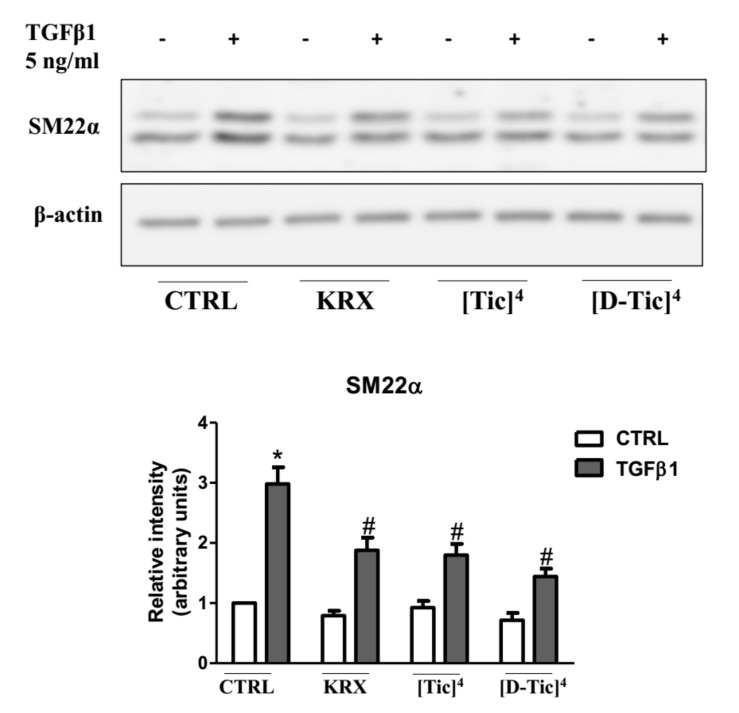
Subconfluent C2C12 myoblasts were pre-incubated with S1P_3_ antagonists KRX-725-II (KRX, 30 μM), [Tic]^4^-KRX-725-II ([Tic]^4^, 30 μM), [D-Tic]^4^-KRX-725-II ([D-Tic]^4^, 30 μM) for 1 h, before being stimulated with 5 ng/mL TGFβ1 for 48 h. The content of SM22α was analyzed by Western blotting in cell lysates. A representative blot is shown. The histograms represent the densitometric analysis of three independent experiments. Data are the mean ± SEM and are reported as protein expression normalized to β-actin, in terms of fold change over control. TGFβ1 significantly increases the expression levels of fibrotic marker SM22α (*, *p* < 0.05). The blockade of S1P_3_ by KRX-725-II, [Tic]^4^-KRX-725-II and [D-Tic]^4^-KRX-725-II significantly inhibits TGFβ1’s effect (#, *p* < 0.05).

**Figure 6:**
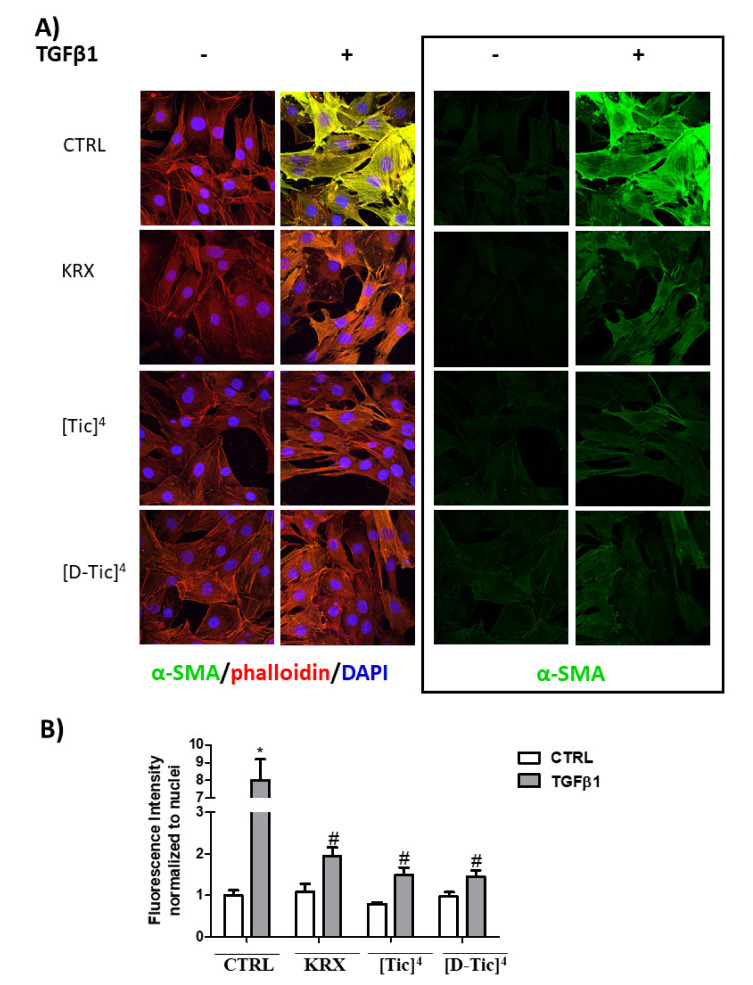
C2C12 cells were seeded on microscope slides and pre-incubated for 1 h with the S1P_3_ antagonists KRX-725-II (KRX, 30 μM) or [Tic]^4^-KRX-725-II ([Tic]^4^, 30 μM) or [D-Tic]^4^-KRX-725-II ([D-Tic]^4^, 30 μM) before being challenged with 5 ng/mL TGFβ1 for 48 h. (**A**) Representative immunofluorescence images of C2C12 myoblasts stained with α-SMA antibody (green), TRITC-labeled phalloidin (red) for F-actin filaments, and DAPI (blue) for nuclei are shown. Images of α-SMA staining only are reported on the right. Magnification × 63. (**B**) Quantification of the intensity of α-SMA fluorescence normalized to DAPI. Data are mean ± SEM of 6 fields quantified in three independent experiments. The blockade of S1P_3_ by KRX-725-II, [Tic]^4^-KRX-725-II and [D-Tic]^4^-KRX-725-II significantly inhibits TGFβ1’s effect (#, *p* < 0.05).

The effect was significant at 30 μM for all three compounds. Compound **VII** was also effective at a 10 µM concentration (Figure 5). To further confirm the efficacy of the three compounds, confocal immunofluorescence experiments were carried out to determine whether KRX-725-II and compounds **V** and **VII** (30 μM) were able to significantly reduce the profibrotic effect of TGFβ1 (5 ng/mL) in C2C12 myoblasts, evaluating the expression level of fluorescein-labeled αSMA. As shown in Figure 6, KRX-725-II and compounds **V** and **VII** inhibited TGFβ1-induced increases in αSMA of 70%, 75% and 85%, respectively.

### 2.4. Molecular Dynamics Simulations

The substitution of tyrosine with the conformationally constrained L-Tic and D-Tic moieties (see compounds **V** and **VII** of Table 1) within the native peptide sequence (KRX-725-II) resulted in potent and selective antagonists for S1P_3_ receptors.

To evaluate the impact of such conformational constraint on the overall flexibility of molecules **V** and **VII** compared to the native peptide KRX-725-II, MD simulations of 1000 ns were performed using the Desmond module implemented in Maestro software.

Given that biochemical and pharmacological studies indicated that the lipid moiety of pepducins has the sole purpose of facilitating the translocation across the lipid bilayer and of anchoring the pepducin in the cytosolic face of the plasma membrane in the vicinity of the target receptor [14,15], we excluded the myristoyl moiety from the MD simulations.

The conformational stability of the peptides during the simulation procedure was examined by calculating the root mean square deviation (RMSD) of the backbone atoms (N, Cα, C) over the entire trajectories, showing an average RMSD for KRX-725-II, **V** and **VII** of 2.4 Å, 2.0 Å and 2.0 Å, respectively. The overall graph of RMSD (Supporting Data, Figure S1a) indicates that the peptides maintained a certain degree of stability, without any rapid changes or jumps throughout the entire trajectory, with compounds **V** and **VII** being the most stable, followed by the native peptide KRX-725-II (least stable).

Furthermore, the radius of gyration (Rg), which is defined as the mass-weighted root mean square distance of all the atoms from their center of mass, was calculated to understand the structural integrity of the peptides. The average Rg values for KRX-725-II, **V** and **VII** were 6.0 Å, 6.3 Å and 5.3 Å, respectively (Supporting Data, Figure S1b). From the figure, it is clear that the modified peptides are much more stable compared to KRX-725-II, with compound **VII** having a more compact structure. In contrast, the native peptide has a highly fluctuating Rg profile, which indicates its more mobile nature in solution.

The hydrogen bonds (H-bonds) are transient interactions that play an important role in the overall stability of protein structures and molecular recognition [16]. To estimate the contribution of intramolecular H-bonds to peptide stability, the total number of H-bonds was computed during MD simulations. The H-bond analysis revealed that **VII** retained between one and four H-bonds until the end of the simulation (Supporting Data, Figure S1c). Conversely, the number of H-bonding contacts was reduced for both KRX-725-II and **V** and was maintained between zero and three until the end of the simulation. This H-bond interaction analysis clearly indicates that compound **VII** exhibits better stability during the whole simulation, which is consistent with the previous Rg results.

Balancing this data, we can conclude that the native peptide is more flexible than the modified peptides, given that it presents a higher RMSD and an unstable Rg. On the other hand, compounds **V** and **VII** appear to be more stable, with compound **VII** having a higher degree of compactness.

To identify the representative conformations of the three peptides occurring during the MD simulations, a clustering analysis was performed, detecting 18, 5 and 8 clusters for KRX-725-II, **V** and **VII**, respectively (Table 2).

The dominant clusters of compounds **V** and **VII** were significantly populated, at 75% and 45.5%, respectively. Conversely, KRX-725-II adopted numerous clusters, with the first five having relative populations of 17%, 12%, 10.5%, 9.5% and 9%, respectively. This analysis indicates that KRX-725-II exhibited larger fluctuations during the MD simulation, thus assuming a more flexible structure with more diversified conformations compared to the modified peptides, which agrees with the previously described results. In addition, analyzing the side-chain orientation of the Tyr4 residue of KRX-725-II in the middle structures from the highest-populated clusters, we noticed that this residue retained considerable orientational freedom, adopting gauche (+) (χ1 = +60°), gauche (−) (χ1 = −60°) and trans (χ1 = 180°) conformations. Conversely, L- and D-Tic showed very limited movement, assuming only a gauche (+) conformation.

**Figure 7:**
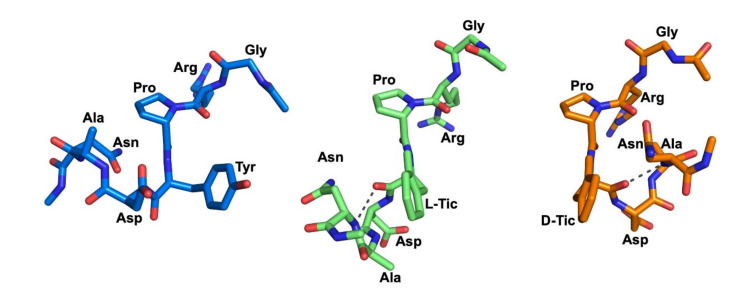
Middle structure from the highest-populated clusters identified during the 1000 ns simulation of KRX-725-II (marine sticks), **V** (green sticks) and **VII** (orange sticks) peptides free in solution.

The middle structures of the highest-populated clusters of the three peptides are shown in Figure 7.

Interestingly, both the middle structures of **V** and **VII** formed a H-bond involving amino acids in positions 4 and 7. In peptide **V**, a backbone H-bond was identified between Asn^7^-NH and L-Tic^4^-CO (3.2 Å). Additionally, one diagnostic tool applicable for the determination of a turn structure is the Cα distance of the residues [17]. In this context, the Cα atoms of Asn^7^ and L-Tic^4^ (compound **V**) were found in close proximity (5.8 Å).

In peptide **VII**, a strong H-bond was formed between the amide proton of Asn^7^ and the carbonyl oxygen of D-Tic^4^ (2.8 Å), while the distance between the Cα atoms of these amino residues measured 5.7 Å. The above data suggest regular β-turns for both peptides **V** and **VII,** comprised of Asn^7^ and L- or D-Tic^4^ residues (Table 3).

**Figure 8:**
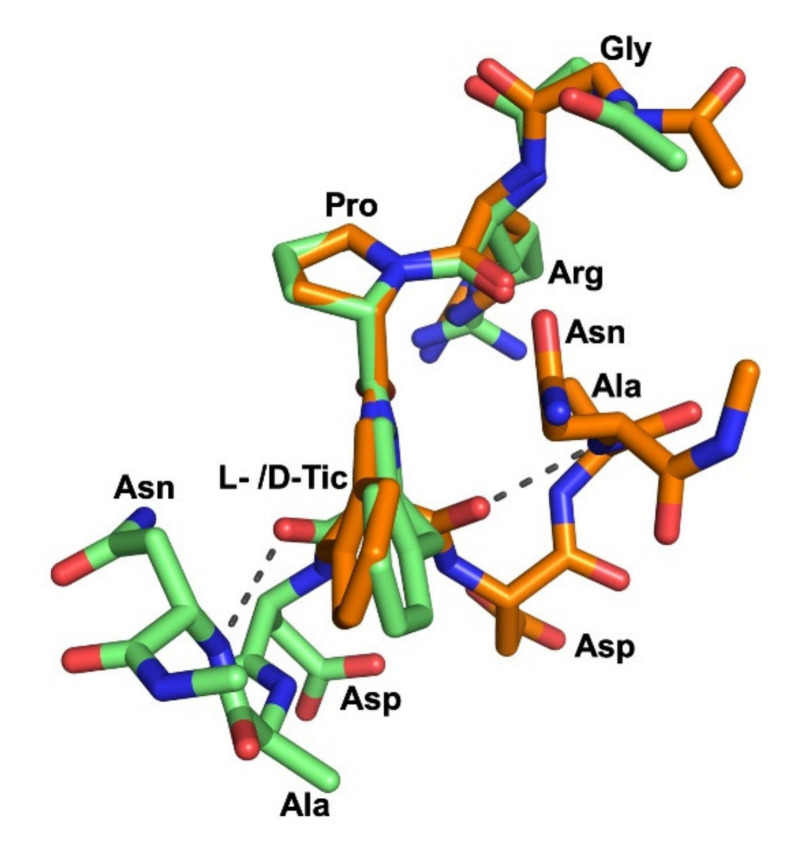
Superposition of representative conformations of peptides **V** (green sticks) and **VII** (orange sticks). The β-turn-like motif observed in two peptides is stabilized by a H-bond (grey dotted line) between Asn^7^-NH and L-Tic^4^-CO.

The middle conformations of **V** and **VII** were compared by superimposing their Gly^1^-Arg^2^-Pro^3^ residues, which adopt a similar backbone orientation (Figure 8).

Most strikingly, this spatial superimposition revealed that the Asp^5^-Ala^6^-Asn^7^ residues with β-turn-like motifs are located on opposite sides of the plane defined by the L- or D-Tic residue, because of the conformational constraints existing in the Tic^4^ residue. This difference in the orientation of the Asp^5^-Ala^6^-Asn^7^ residues folding in a β-turn-like motif may explain, in structural terms, the selective S1P_3_ antagonism of **V** and **VII** in comparison to the unselective antagonist KRX-725-II. In practice, the flexibility of KRX-725-II seems to be high enough for adaptation to the binding regions of the individual receptor subtypes S1P_1_ and S1P_3_, whereas peptides **V** and **VII** possess a highly constrained D- or L-Tic residue that prevents the pharmacophore from interacting properly with the binding pocket of the S1P_1_ receptor, therefore leading to S1P_3_ selectivity.

## 3. Discussion

The S1P_1_ and S1P_3_ receptors show the highest homology degree within the S1PR family. They have about 50% identical and >70% similar amino acids [18]. The main difference in their binding pockets derives from the S1P_1_ Leu276 and S1P_3_ Phe263 side chains, thus leading to great difficulty in obtaining selective orthosteric ligands. For this reason, we focused our attention on pepducins, which can work as allosteric ligands that bind sites alternative to the orthosteric. This approach provides certain advantages in terms of specificity and selectivity [19].

The molecules here described (compounds **I-IX**, Table 1) have been designed to improve the selectivity profile of KRX-725-II, the first pepducin S1P_3_ antagonist to be described [10]. The first screening allowed us to make some considerations on the structure–activity relationships. Compound **I**, [D-Tyr]^4^-KRX-725-II, retains its antagonistic activity (Figure 1A), indicating that the interaction with the target is not stereospecific. The fact that compounds **I** and **II**, as opposed to the other aromatic substituted derivatives (such as **III** and **IV**), work as antagonists, even if unselective, suggests the need for the presence of a weakly acid group. This group is probably involved in the formation of a hydrogen bridge. The same condition is not satisfied for compound **VI**, most likely for steric reasons. Nevertheless, when sterically constrained cyclic amino acids are introduced, this requirement is no longer necessary for the preservation of the antagonistic activity. Indeed, compound **V**, in addition to being active, is also selective towards the S1P_3_ receptor (Figure 1, Figure 2 and Figure 3). This has been demonstrated using both allosteric (KRX-725 and KRX-722; Figure 1) and orthosteric selective agonists (CYM5541 and SEW2871; Figure 2).

Cyclically constrained aromatic amino acids, such as Tic, have been widely used to control the χ1 and χ2 space to discover more potent bioactive peptidomimetics ligands [20,21,22,23,24,25]. The Tic residue introduces not only a restricted orientation of the aromatic side chain, but also a conformational constraint into the peptide backbone via the pipecolic acid bridge, thus significantly minimizing the flexibility of the native peptide. Interestingly, the substitution of tyrosine with both the conformationally constrained L-Tic (compound **V**) and D-Tic (compound **VII**) moieties within the native peptide sequence (KRX-725-II) resulted in potent and selective antagonists for S1P_3_ receptors. Even in this case, the interaction is not stereospecific. The so-identified derivatives have also been also evaluated for their ability to reduce the profibrotic action of the cytokine TGFβ1 in C2C12 murine myoblasts, previously shown to depend upon the S1P_3_ receptor [7]. Interestingly, although the three compounds were all effectively inhibited at 30 µM, the increased expression of fibrotic marker SM22α induced by TGFβ, compound **VII**, was already active at 10 µM. Moreover, compound **V** and compound **VII** caused a more marked inhibition of TGFβ1-induced increases in αSMA in comparison to KRX-725-II.

## 4. Materials and Methods

### 4.1. Chemistry

#### 4.1.1. Chemicals

Protected α-amino acids and Rink Amide MBHA-resin were purchased from Bachem (Bubendorf, Switzerland) and Merck-Novabiochem (Darmstadt, Germany). The protecting groups for the α-amino acids were as follows: Fmoc for α-amino groups; *t*Bu for Tyr; O*t*Bu for Asp; Boc for Lys and Trp; Pbf for Arg; and trityl (Trt) for Asn. Solvents and other chemicals for peptide synthesis and purification were purchased from Sigma–Aldrich (Milan, Italy). Mass spectra of the final products were derived on an API 2000 Applied Biosystem mass spectrometer. RP-HPLC preparative purification was routinely performed on a Shimadzu system equipped with a Shimadzu multiwavelength detector on a Phenomenex Kinetex XB-C18 column (5 μm, 21.2 × 250 mm). The operational flow rate was 30 mL/min. The homogeneity of the products was assessed by analytical RP-HPLC using a Phenomenex Kinetex XB-C18 column (5 μm, 4.6 × 250 mm). The column was connected to a Rheodyne model 7725 injector, a Shimadzu-10 ADsp HPLC system and a Shimadzu SPD-20 A/SPD-20 AV UV-VIS detector set to 220 nm.

#### 4.1.2. Peptide Synthesis

Peptides I–IX were synthesized according to the solid phase approach using the standard Fmoc methodology in a manual reaction vessel and in a stepwise fashion [26]. For each peptide, 0.5 g (0.35 mequiv) of Rink Amide MBHA resin was used. The resin was swelled in DMF for 1 h and packed in the reaction vessel. Each Fmoc–amino acid was coupled in a 3-fold excess, via TBTU/HOBt/DIPEA, except for the first amino acid, which was used at a 5-fold excess and coupled via DIPCDI/HOBt. The Fmoc-protecting groups were removed by treating the protected peptide resin with a 25% solution of piperidine in DMF (45 min). The peptide resin was washed three times with DMF, and the next coupling step was initiated in a stepwise manner. Myristoyl glycine was coupled in a 5-fold excess as an N-terminal residue via TBTU/HOBt/DIPEA using a mixture of DCM/NMP (1/1) as a reaction solvent. All reactions were performed under nitrogen bubbling to stir the reaction mixtures and avoid the oxidation of methionine-containing peptides. After the completion of the synthesis, the peptide resin was washed with DMF (3×) and DCM (4×), and then dried in vacuo. Each protected peptide was cleaved from the resin, and the amino acid side chains were simultaneously deprotected by treatment with a mixture of TFA/DCM/TIS/anisole (90/5/3/2) for 2 h at room temperature under nitrogen atmosphere. The resin was removed by filtration, and the crude peptide was recovered by precipitation with cold anhydrous ethyl ether to give a white powder, which was purified by RP-HPLC on a preparative Phenomenex Kinetex XB-C18 column using a gradient of CH_3_CN in 0.1% aqueous TFA (from 30% to 60% in 30 min) at a flow rate of 30 mL/min. The final product was obtained by lyophilization of the appropriate fractions after removal of the CH_3_CN by rotary evaporation. Analytical RP-HPLC (two-solvent system: A–0.05% TFA (*v/v*) in water and B—0.05% TFA (*v/v*) in acetonitrile eluted in a linear gradient from 30 to 60% B over 20 min, UV detection at 220 nm, flow rate 1 mL/min) indicated a purity >98% and the correct molecular weights were confirmed by ESI-MS. The analytical data and the operative conditions employed for assessing the homogeneity of the compounds are reported in Table 1.

### 4.2. Pharmacology

#### 4.2.1. Aorta Rings Assay

Male mice Cd-1 (20–25 g, Harlan, Italy) were housed in an environment with controlled temperature (21–24 °C) and lighting (12:12 light–darkness cycle). Standard chow and drinking water were provided ad libitum. A period of 7 days was allowed for the acclimatization of rats before undertaking any experimental manipulation. All the experiments were conducted following the principles of laboratory animal care (law no. 86/609/CEE), as well as specific national law (no. 116/1992). Animals were anaesthetized by inhalation of isoflurane, and after exsanguinations, the thoracic aorta was removed, cleaned of adherent connective tissue, and cut into rings of 3 mm in length. Rings were mounted under 1.0 g of tension in 2.5 mL organ baths containing Krebs salt solution of the following composition (in millimolar): NaCl, 118.4; KCl, 4.7; MgSO_4_, 1.2; CaCl_2_, 1.3; KH_2_PO_4_, 1.2; NaHCO_3_, 25.0; glucose, 11.7. The solution was maintained at 37 °C and bubbled with 95% O_2_–5% CO_2_ (pH 7.4). Developed tension was measured using an isometric force transducer (Basile, Italy) connected to a recorder. Rings were allowed to equilibrate for 60 min, and the Krebs solution was replaced every 15 min. In each experiment, aortic rings were firstly challenged with PE (10^−6^ M) until the responses were reproducible. To verify the integrity of the endothelium, the acetylcholine cumulative concentration–response curve (10^−8^ − 3 × 10^−5^ mol/L) was assessed for the PE-contracted rings. Vessels that relaxed less than 85% were discarded. Aortic rings were contracted with PE (10^−6^ mol/L), and once a plateau was reached in the presence of the synthesized compounds, cumulative concentration–response curves with KRX-725 (a pepducin derived from the second intracellular loop of S1P_3_ that triggers specifically the S1P_3_ receptor), KRX-722 (a pepducin derived from the second intracellular loop of S1P_1_ that triggers specifically the S1P_1_ receptor), SEW2871 (S1P_1_ agonist) or CYM5541 (S1P_3_ agonist) (10^−8^ – 3 × 10^−5^ mol/L) was derived. Relaxations have been expressed as the percentage of contraction. Relaxations were determined for each concentration–response curve by nonlinear regression analysis.

#### 4.2.2. Materials

All biochemicals, cell culture reagents, DMEM, fetal calf serum, protease inhibitor cocktail, α-smooth muscle actin (αSMA) antibody, bovine serum albumin (BSA), 4′,6-diamidino-2-phenylindole dihydrochloride (DAPI), enhanced chemiluminescence reagents, and TRITC-phalloidin were purchased from Merck Millipore (Burlington, MA, USA). Mouse skeletal muscle C2C12 cells were obtained from the American Type Culture Collection (Manassas, VA, USA), and recombinant transforming growth factor (TGF) β1 was obtained from PeproTech (London, UK). The anti-SM22 alpha antibody was from Everest Biotech (Oxford, UK). Secondary antibodies conjugated to horseradish peroxidase and monoclonal anti-β-actin antibodies were obtained from Santa Cruz Biotechnology (Santa Cruz, CA, USA). Fluorescein-conjugated horse anti-mouse secondary antibodies were obtained from Vector Laboratories (Burlingame, CA, USA).

#### 4.2.3. Cell Culture and Agonist Treatment

Murine C2C12 myoblasts were routinely grown in DMEM supplemented with 10% fetal calf serum, 2 mM L-glutamine, 100 U/mL penicillin, and 100 μg/mL streptomycin at 37 °C in 5% CO_2_ [27]. When requested, cells were incubated with the S1P_3_ antagonists KRX-725-II, [Tic]^4^-KRX-725-II and [D-Tic]^4^-KRX-725-II at the indicated concentrations, 1 h before being challenged with 5 ng/mL TGFβ1 for 48 h.

#### 4.2.4. Western Blot Analysis

C2C12 cells were lysed for 30 min at 4 °C in a buffer containing 50 mM Tris, pH 7.5, 120 mM NaCl, 1 mM EDTA, 6 mM EGTA, 15 mM Na_4_P_2_O_7_, 20 mM NaF, 1% Nonidet and protease inhibitor cocktail (1.04 mM AEBSF, 0.08 μM aprotinin, 0.02 mM leupeptin, 0.04 mM bestatin, 15 μM pepstatin A, and 14 μM E-64), essentially as described in Bernacchioni et al., (2018) [28]. To prepare total cell lysates, cell extracts were centrifuged for 15 min at 10,000× *g* at 4 °C. Proteins from cell lysates were resuspended in Laemmli’s SDS sample buffer. Samples were subjected to SDS-PAGE before the transfer of proteins to PVDF membranes. Membranes were incubated overnight with the primary antibodies against α-SMA, SM22 alpha and β-actin at 4 °C and then with specific secondary antibodies for 1 h at room temperature. Bound antibodies were detected by chemiluminescence.

#### 4.2.5. Cell Immunofluorescence

Cells were seeded on microscope slides and then pre-incubated with S1P_3_-specific antagonists before being challenged with 5 ng/mL TGFβ1 for 48 h. Immunofluorescence was assessed as described in Bruno et al., (2018) [29]. Briefly, cells were fixed in 2% paraformaldehyde in PBS for 20 min and permeabilized in 0.1% Triton X-100-PBS for 30 min. The cells were then blocked in 3% BSA for 1 h, and incubated with anti-α-SMA antibody for 2 h and fluorescein-conjugated anti-mouse secondary antibody for 1 h. To stain nuclei, the specimen was incubated with 250 nM DAPI in PBS for 15 min, then washed with PBS and incubated with TRITC-phalloidin for 1 h. Images were obtained using a Leica SP8 laser scanning confocal microscope (Leica Microsystems GmbH, Wetzlar, Germany) with a 63× objective.

#### 4.2.6. Statistical Analysis

Densitometric analysis of the Western blotting bands was performed using ImageJ software (https://imagej.nih.gov/ij/ access on 17 August 2021). Graphical representations were created using GraphPad Prism 6.0 (GraphPad Software, San Diego, CA, USA). Statistical analysis was performed using one-way or two-way ANOVA followed by Bonferroni’s post hoc test. Asterisks indicate statistical significance, as indicated in figure legends.

### 4.3. Computational Chemistry

#### 4.3.1. Molecular Dynamics Simulation

Peptides KRX-725-II, **V** and **VII** were sketched using the Molecular Builder module in Maestro (Schrödinger, LLC, New York, NY, USA, 2021), capping the N-terminus with an acetyl group (ACE) and the C-terminus with an N-methyl amide group (NMA). The peptides were then optimized using Macromodel (Schrödinger, LLC, New York, NY, USA, 2021), employing the MMFFs force field with 1000 steps of steepest descent and 500 steps of truncated Newton conjugate gradient algorithms.

MD simulations were performed using the Desmond Multisim protocol implemented in Maestro. Each peptide lacking the myristoyl moiety was solvated with TIP3P water molecules in an orthorhombic box of 10Å × 10Å × 10Å in size. The number of water molecules around each peptide was found to be as follows: KRX-725-II—1308; V—1405; VII—1221. No counterions were included.

The systems were relaxed before the simulation by using the protocol implemented in Desmond, and then they were simulated for 1000 ns via NTP ensemble using the Nose-Hoover thermostat [30] to maintain a constant temperature of 350 K and isotropic Martyna–Tobias–Klein barostat [31] to maintain the pressure at 1 atm. The simulation temperature was slightly higher than room temperature, which aided in avoiding kinetic traps and allowing us to probe the stabilities and dynamics of pepducins more quickly during the simulation. The short-range Coulombic interactions were analyzed with a cut-off value of 9.0 Å using the short-range method. A time-reversible reference system propagator algorithm (RESPA) integrator was used with a time step of 2.0 fs [32].

#### 4.3.2. Analysis of Molecular Dynamics Trajectory

The MD trajectories were analyzed using the Simulation Event Analysis and Simulation Interactions Diagram tools available with the Desmond module. The root mean squared deviation (RMSD) data for KRX-725-II, **V** and **VII** were calculated from the simulation interaction diagram, whereas the numbers of intramolecular H-bonds and Rg were obtained from the simulation event analysis.

The Desmond trajectory clustering tool was used to select representative structures from the simulations. The backbone RMSD matrix was used as the structural similarity metric, while hierarchical clustering with average linkage was selected as the clustering method. The merging distance cutoff was set to be 1.7 Å. The centroid structure (i.e., the structure with the largest number of neighbors in the structural family) was used to represent the structural family. Figures were generated using Pymol (Schrödinger, LLC, New York, NY, USA, 2021).

## 5. Conclusions

Overall, these findings suggest the increased efficacy of the two compounds **V** and **VII** in comparison to KRX-725-II, in agreement with their augmented stability/compactness highlighted by molecular dynamics simulations. In conclusion, these selective S1P_3_ antagonists will be useful to better define the role of S1P_3_ in fibrosis, as well as to develop a novel therapeutic strategy to treat inflammation-related fibrotic diseases.

## Figures and Tables

**Table 1:** Chemical data for compounds **I-IX**.

	Compound	t_R_ (min) ^a^	MW ^b^	
			Calcd	Found
**KRX-725**	Myristoyl-GMRPYDANKR-NH_2_	13.751	1416.7	1417.1
**KRX-725-II**	Myristoyl-G-RP**Y**DAN-NH_2_	14.642	1001.2	1001.2
**I**	[D-Tyr]^4^-KRX-725-II	13.967	1001.2	1001.2
**II**	[Trp]^4^-KRX-725-II	16.156	1024.2	1024.4
**III**	[Phe]^4^-KRX-725-II	16.567	985.2	985.7
**IV**	[p-F-Phe]^4^-KRX-725-II	16.509	1003.2	1003.6
**V**	[Tic]^4^-KRX-725-II	17.690	997.2	997.9
**VI**	[(4-NHSO_2_CH_3_)Phe]^4^-KRX-725-II	17.562	1078.3	1078.2
**VII**	[D-Tic]^4^-KRX-725-II	17.705	997.2	997.8
**VIII**	[Pip]^4^-KRX-725-II	14.886	949.15	949.8
**IX**	[Pro]^4^-KRX-725-II	15.625	935.12	935.7

^a^ Retention time determined by RP-HPLC, obtained using the following conditions: reversed phase Phenomenex Kinetex XB-C18 column (5 μm, 4.6 × 250 mm) and a two solvents system (A—0.05% TFA (*v/v*) in water and B—0.05% TFA (*v/v*) in acetonitrile) eluted in a linear gradient from 30 to 60% B over 20 min, UV detection at 220 nm, flow rate 1 mL/min. ^b^ *m/z* values measured by ESI-MS.

**Table 2:** Clustering analysis results obtained with an RMSD cut-off value of 1.7 Å.

	Cluster 1	Cluster 2	Cluster 3	Cluster 4	Cluster 5
KRX-725-II (18)	17%	12%	10.5%	9.5%	9%
Compound **V** (5)	75%	14%	5.5%	4%	1.5%
Compound **VII** (8)	47.5%	33.5%	6%	5.5%	3.5%

The total number of clusters obtained from the analysis of each peptide’s trajectory is indicated in parentheses in the first column. The relative population of the most representative frame (the centroid) is shown for the five highest-populated clusters.

**Table 3:** Torsion angles parameters, H-bond distance and Cα–Cα distance between Asn^7^ and L- or D -Tic^4^ residues.

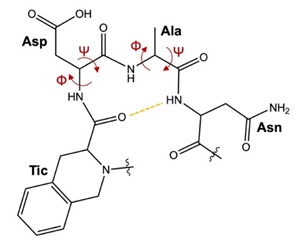						
	**Asp**		**Ala**			
	**Φ**	**Ψ**	**Φ**	**Ψ**	**H-Bond Distance**	**L- or D-Tic/Asn Cα Distance**
Compound **V**	−73.9°	−4.9°	−99.3°	22.8°	3.2 Å	5.8 Å
Compound **VII**	−59.4°	−11.6°	−76.9°	−28.1°	2.8 Å	5.7 Å

## Data Availability

Not applicable.

## Supplementary Materials

The following are available online at https://www.mdpi.com/article/10.3390/ijms22168861/s1.
