# Supplementary material for: Antagonizing S1P3 Receptor with Cell-Penetrating Pepducins in Skeletal Muscle Fibrosis
Source: Int J Mol Sci. 2021 Aug 17;22(16):8861. doi: 10.3390/ijms22168861 (PMC8396189; doi:10.3390/ijms22168861)
Supplement: Supplementary file 1 [file ijms-22-08861-s001.zip › ijms-1323291-supplementary.pdf]

Antagonizing $S1P_3$ Receptor with Cell-Penetrating Pepducins in Skeletal Muscle Fibrosis

Angela Corvino [1], Ida Cerqua [1], Alessandra Lo Bianco [1], Giuseppe Caliendo [1], Ferdinando Fiorino [1], Francesco Frecentese [1], Elisa Magli [1], Elena Morelli [1], Elisa Perissutti [1], Vincenzo Santagada [1], Giuseppe Cirino [1], Elisabetta Granato [1], Fiorentina Roviezzo [1], Elisa Puliti [2], Caterina Bernacchioni [2], Antonio Lavecchia [1], Chiara Donati [2] and Beatrice Severino [1,*,†]

[1] Department of Pharmacy, School of Medicine, University of Naples «Federico II», Via D. Montesano, 49, 80131 Napoli, Italy; angela.corvino@unina.it (A.C.); ida.cerqua@unina.it (I.C.); alessandra.lobianco@unina.it (A.L.B.); caliendo@unina.it (G.C.); fefiorin@unina.it (F.F.); frecente@unina.it (F.F.); elisa.magli@unina.it (E.M.); elena.morelli@unina.it (E.M.); perissut@unina.it (E.P.);
santagad@unina.it (V.S.); cirino@unina.it (G.C.); elisabetta.granato@unina.it (E.G.); roviezzo@unina.it (F.R.); antonio.lavecchia@unina.it (A.L.)

[2] Department of Experimental and Clinical Biomedical Sciences "Mario Serio", University of Florence,
Viale GB Morgagni 50, 50134 Firenze, Italy; elisa.puliti@unifi.it (E.P.); caterina.bernacchioni@unifi.it (C.B.); chiara.donati@unifi.it (C.D.)

* Correspondence: bseverin@unina.it; Tel.: +39-081-679-828

† Co-last authors.

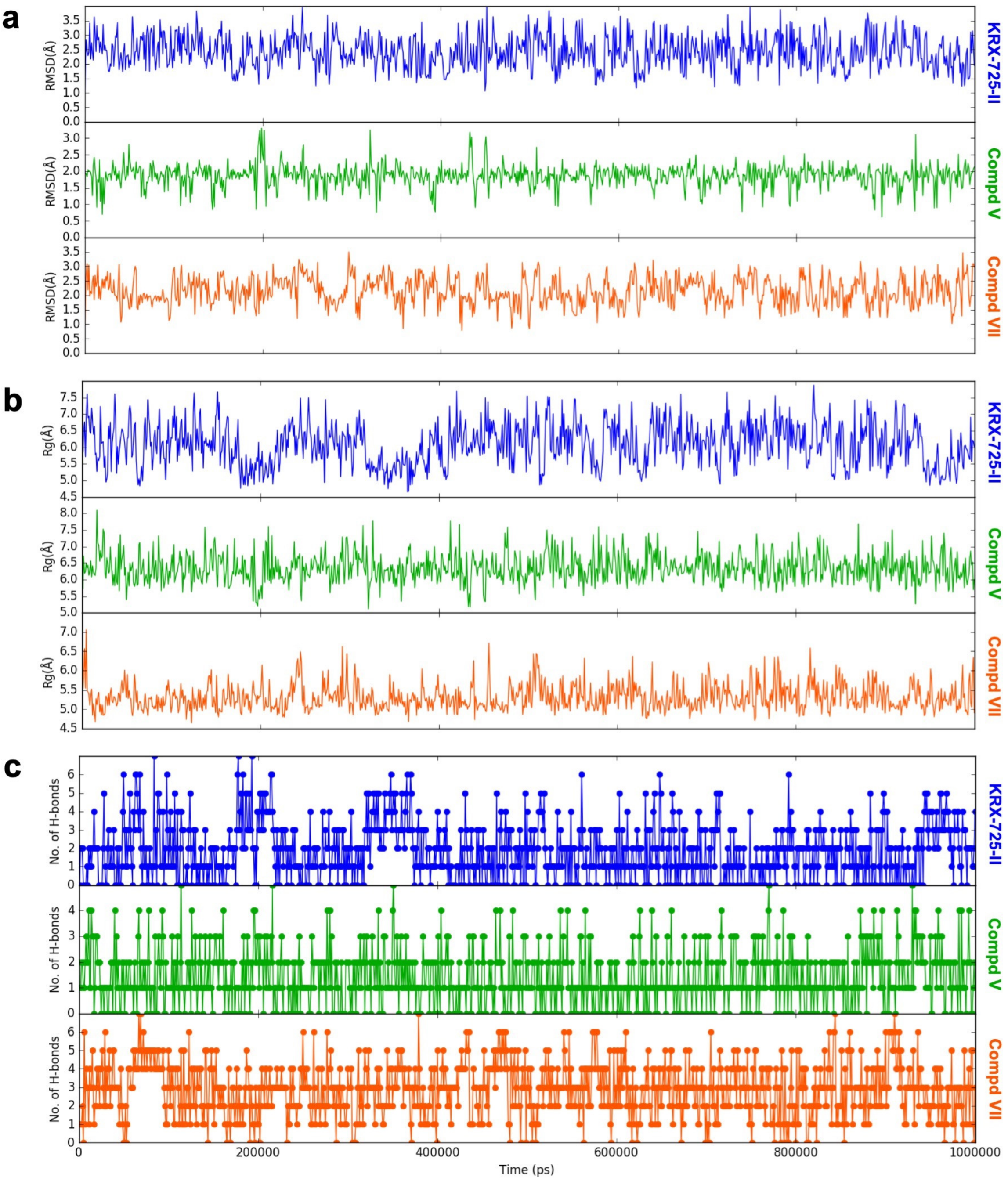

Figure S1. (a) Time evolution of the backbone RMSD compared to frame 0 for **KRX-725-II** (blue), compound **V** (green) and compound **VII** (orange) during 1000 ns of MD. **(b)** Variation of the Rg with time for **KRX-725-II** (blue), **V** (green) and **VII** (orange). **(c)** Total number of intramolecular H-bonds formed by **KRX-725-II** (blue), **V** (green) and **VII** (orange) during the entire simulation period.
